# Current perspectives and updates on transfusion strategies in critically ill anemic patients

**DOI:** 10.3389/fmed.2026.1762664

**Published:** 2026-03-10

**Authors:** Haowei Yang, Feifei Li, Zhenghua Zhu, Lining Si, Yi Liu, Songbei Li, Yang Liu, Hua Cai, Yuxin Fan, Yuxuan Zhang, Chun Pan, Sen Lu, Hongli He

**Affiliations:** 1 School of Medicine, University of Electronic Science and Technology of China, Chengdu, China; 2 Department of Respiratory and Critical Care Medicine, The Second People’s Hospital of Kunming, Kunming, China; 3 Department of Critical Care Medicine, Affiliated Hospital of Qinghai University, Xining, China; 4 Department of Intensive Care Unit, Affiliated Hospital of Chengdu University, Chengdu, China; 5 Department of Critical Care Medicine, Sichuan Provincial People’s Hospital Pujiang Hospital/Pujiang County People’s Hospital, Chengdu, China; 6 School of Medical and Life Sciences, Chengdu University of Traditional Chinese Medicine, Chengdu, China; 7 Department of Critical Care Medicine, Sichuan Provincial People’s Hospital, Affiliated Hospital of University of Electronic Science and Technology of China, Chengdu, China

**Keywords:** blood transfusion, intensive care units, liberal transfusion, restrictive transfusion, transfusion threshold

## Abstract

Anemia is a frequently encountered condition in critically ill patients, and red blood cell transfusion serves as a critical therapeutic intervention aimed at restoring hemoglobin levels and improving tissue oxygenation. However, the optimal threshold for transfusion remains a subject of controversy, particularly regarding the comparison between restrictive (Hb 7–9 g/dL) and liberal (Hb > 9 g/dL) strategies. However, we should also consider a third option. This method focuses on the individual patient. Doctors adjust care based on the patient’s specific symptoms and other health problems. The selection of the appropriate strategy is contingent upon specific clinical conditions, such as sepsis, acute coronary syndrome, or neurological injury. Current evidence and clinical guidelines predominantly favor a restrictive transfusion in hemodynamically stable patients, whereas a liberal strategy may benefit those with underlying cardiovascular disease. This review synthesizes recent trial data and guideline recommendations to facilitate evidence-based and individualized transfusion decision-making in the ICU, aiming to optimize the balance between risks and benefits across diverse critical care settings.

## 1 Introduction

Anemia is a highly prevalent condition among critically ill patients, defined by the World Health Organization (WHO) as hemoglobin falling levels below 12 g/dL in women and 13 g/dL in men. In 2021, the global prevalence of anemia across all age groups was reported to be 24.3%, with severe, moderate, and mild anemia constituting 0.9%, 9.3%, and 14.1% of cases, respectively (1). Patients admitted to the intensive care unit (ICU) present with a significantly higher severity of illness compared to those in general hospital ward. In the ICU, approximately 95% of patients are estimated to develop anemia by Day 3 of their admission, a condition that typically persists throughout their hospital stay, irrespective of transfusion status (2–4).

While the concept of blood transfusion can be traced back to 43 BC in the Book VII of Ovid’s Metamorphoses, the first documented human transfusion was performed in the 19th century by James Blundell (5). Blood transfusion serves as a critical intervention for hemorrhage and a fundamental therapeutic strategy for anemia in critically ill patients, rapidly restoring hemoglobin levels and improves tissue oxygenation (6). Approximately 30%–50% of ICU patients receive red blood cell (RBC) transfusions during their hospitalization (7). However, this intervention is also associated with (8), including increased susceptibility to infections and organ injury (9). Therefore, doctors must follow proven rules for blood transfusions. These rules come from strong evidence. This approach helps balance the benefits and the risks. It also lowers the chance of harmful side effects for the patient (10). Clinicians should avoid over-reliance on static hemoglobin thresholds, instead prioritizing comprehensive clinical judgment. Assessment should include the presence of physiological symptoms and the patient’s hemodynamic stability. The trend of the blood levels matters too. This method fits the goal of personalized care (11).

Current transfusion guidelines derived from non-critically ill populations may prove inadequate for guiding clinical decision-making in hemodynamically compromised critically ill patients. Although numerous randomized controlled trials have investigated transfusion strategies in critical care, substantial controversy persists regarding the optimal hemoglobin threshold–particularly concerning the choice between a restrictive approach and a more liberal transfusion strategy (12–14). This comprehensive review synthesizes contemporary evidence and guideline recommendations regarding the indications and thresholds for blood product administration in critically ill patients. Consequently, this review aims to establish a practical clinical framework to facilitate transfusion-related decision-making for ICU practitioners.

### 1.1 Search strategy and selection criteria

To ensure a comprehensive review of current evidence, we conducted a systematic literature search using PubMed, Embase, and the Cochrane Library databases from inception up to January 2025. The search strategy employed a combination of Medical Subject Headings (MeSH) and free-text terms, including “red blood cell transfusion,” “hemoglobin threshold,” “restrictive versus liberal,” “critical care,” “intensive care unit,” and “patient blood management.”

We prioritized high-quality evidence, specifically randomized controlled trials (RCTs), systematic reviews, meta-analyses, and international clinical practice guidelines. Given the rapid evolution of transfusion medicine, special emphasis was placed on high-impact trials and updates published between 2023 and 2024 (e.g., MINT, TRAIN and updated AABB/ESICM guidelines). Reference lists of identified articles were manually reviewed to ensure inclusion of relevant historical context. The search was limited to articles published in the English language.

## 2 Common causes and mechanisms of anemia

**FIGURE 1:**
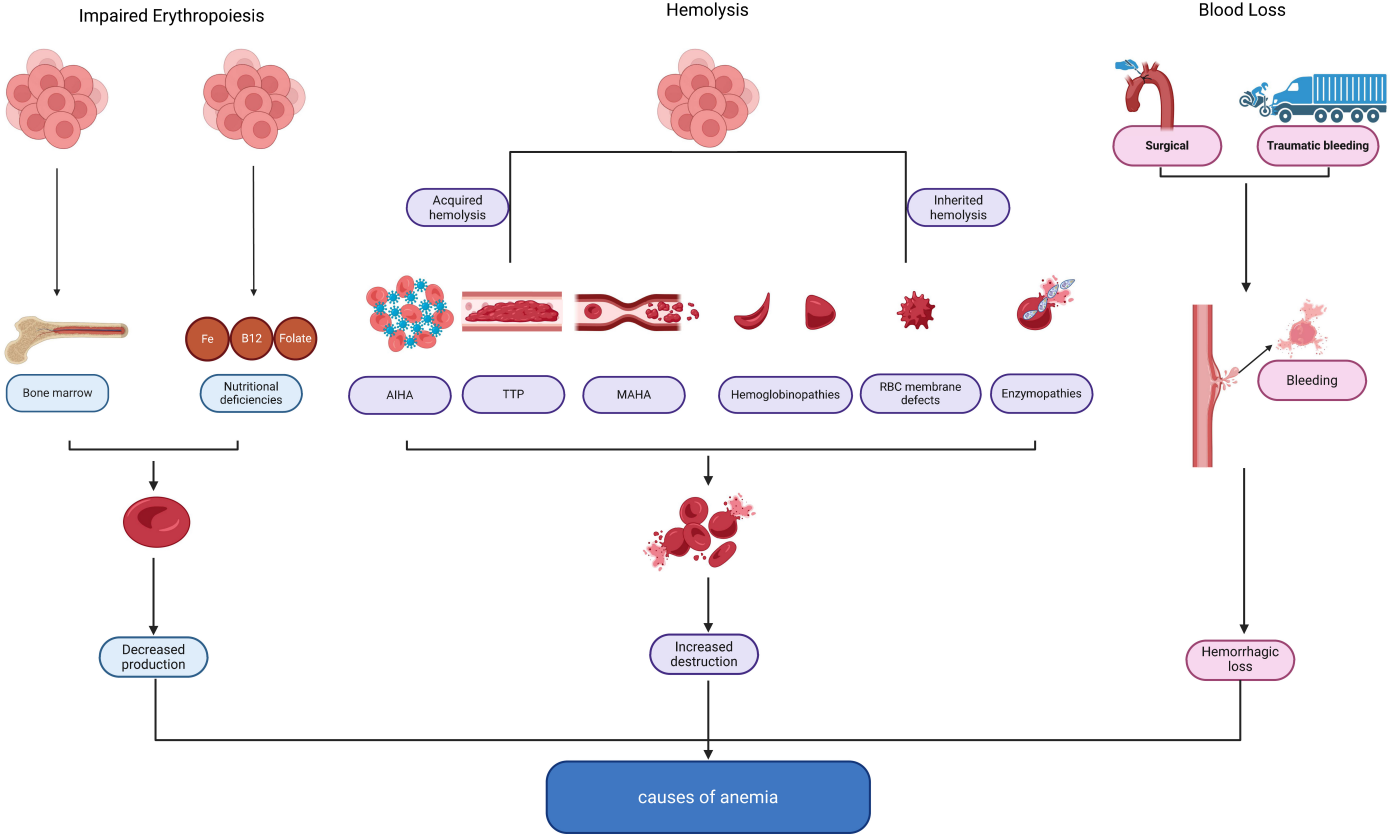
Etiological classification and pathophysiology of anemia in the ICU. AIHA, autoimmune hemolytic anemia; TTP, thrombotic microangiopathies; MAHA, mechanical hemolysis.

Anemia is etiologically classified into three principal mechanisms (15): impaired erythropoiesis, accelerated hemolysis, and blood loss (Figure 1). Impaired erythropoiesis is frequently associated with nutritional deficiencies (e.g., iron, vitamin B12) or bone marrow disorders. Accelerated hemolysis typically results from congenital disorders (e.g., thalassemia, sickle cell disease) or acquired etiologies (e.g., autoimmune hemolysis). Hemorrhagic loss, characterized by an acute reduction in circulating erythrocyte mass, manifests across a diverse spectrum of clinical settings. Distinguishing these fundamental etiologies from contributing factors is critically important in specific patient populations.

In the context of critical illness, the etiology of anemia is frequently multifactorial. Underlying pathologies (such as sepsis, hepatic impairment, and renal dysfunction) can induce anemia of inflammation, a condition driven by proinflammatory cytokines (including IL-1, IL-6, TNF-α, and IFN-γ) and characterized by impaired iron utilization and suppressed erythropoiesis (16).

Concurrently, recurrent iatrogenic interventions–such as diagnostic phlebotomy and the utilization of extracorporeal circuits (e.g., CRRT, ECMO) (17–19)-significantly exacerbate blood loss. Furthermore, transfusion therapy itself may alter the pathophysiology of anemia through mechanisms including hemolytic reactions and the suppression of endogenous erythropoiesis secondary to iron overload (20).

Chronic anemia, frequently referred to as anemia of inflammation, predominantly arises from persistent inflammatory states, chronic kidney disease, or other underlying pathologies (21). This type of anemia is commonly identified in patients prior to ICU admission, surgical procedures, or traumatic injuries. Management necessitates a distinct therapeutic strategy compared to anemia resulting from acute bleeding or hemolysis, prioritizing the treatment of the underlying condition rather than depending exclusively on transfusion assistance.

While blood loss and chronic inflammation remain the predominant etiologies, clinicians must maintain vigilance regarding drug-induced anemia–specifically drug-induced hemolytic anemia (DIHA) (22, 23)-particularly when involving agents such as beta-lactam antibiotics. This condition is primarily classified into two distinct categories: immune-mediated and non-immune-mediated mechanisms (Table 1). Upon suspicion of drug-induced anemia, the immediate discontinuation or substitution of the offending agent constitutes the most effective and definitive therapeutic intervention (22–25).

**TABLE 1:** Common pharmacological agents associated with anemia in the intensive care unit.

Drug category	Common agents	Notes/key features
Hapten/drug-adsorption	Penicillin G Cephalothin Cefazolin Tetracycline	This type of mechanism typically induces extravascular hemolysis.
Immune complex/innocent bystander	Ceftriaxone Quinidine Rifampin	Ceftriaxone-induced hemolysis in children is often acute and severe.
Autoantibody mechanism	Levodopa Fludarabine Procainamide	Fludarabine is the most common drug causing autoimmune hemolytic anemia in recent years
Non-immunologic protein adsorption	Sulbactam Tazobactam Clavulanate Cisplatin Cefotetan	It may be related to plasma IgG levels in patients with hypergammaglobulinemia.
Special/mixed mechanism drugs	Cefotetan Piperacillin	Cefotetan is the most common drug causing DIIHA in recent years, often resulting in fatal hemolysis
Oxidant-induced hemolysis	Primaquine Salicylazosulfapyridine Pamaquine	
Direct toxicity	Phenylhydrazine Ribavirin	

## 3 Impact of anemia

Hemoglobin, the primary oxygen-carrying protein complex within erythrocytes, serves as a fundamental biomarker for evaluating the severity of anemia. Variations in hemoglobin levels reflect both the severity of underlying pathologies (such as cancer or cardiovascular disease) and the cumulative impacts of therapeutic interventions (including surgical blood loss or gastrointestinal bleeding), all of which may independently or synergistically influence clinical outcomes (26–28). As a multifunctional molecule, hemoglobin plays a crucial role in oxygen transport, cellular signaling, and catalytic processes (29). Its physiological roles functions are delineated into four major domains: (1) oxygen to peripheral tissues; (2) regulation of cardiovascular and immune functions via nitric oxide binding; (3) maintenance of acid-base balance through buffering capacity; and (4) contribution to thermoregulation. Additionally, hemoglobin modulates metabolic activity by influencing the function of erythrocyte membrane proteins. A reduction in hemoglobin concentration precipitates impaired oxygen delivery, microcirculatory dysfunction, and diminished buffering capacity, all of which ultimately lead to adverse clinical outcomes.

Although anemia represents a common and readily diagnosable condition, its prognostic implications within the ICU setting remain complex and multifactorial. Research indicates that patients with anemia undergoing elective general surgery exhibit significantly elevated mortality rates compared to their non-anemic counterparts (30), with the severity of anemia closely correlating with the risk of mortality (31).

Postoperative anemic patients exhibited approximately a 1.25-fold higher incidence of Major Adverse Cardiac and Cerebrovascular Events (MACCEs) at 1 year compared to their non-anemic counterparts (95% vs. 76%) (9). Preoperative anemia, particularly among cardiac surgery patients, was associated with significantly increased rates (17.7% vs. 7.6%) and severity of postoperative acute kidney injury (AKI), alongside a prolonged length of hospital stay (extending by 0.9 days) and a 3.6 percentage-point increase in reoperation rates (32). Consequently, the safe and effective elevation of hemoglobin levels has emerged as a pivotal strategy for enhancing outcomes in critically ill populations.

## 4 The advancement of transfusion and its effect on patient outcomes

In the setting of acute hemoglobin reduction, transfusion of packed red blood cells serves as a critical intervention for maintaining adequate oxygen delivery to vital organs, particularly the heart and brain (33). Conversely, the care of chronic inflammatory anemia (also known as anemia of inflammation, AI) predominantly emphasizes the regulation of the underlying inflammatory process (21), necessitating thorough preoperative assessment to mitigate perioperative bleeding risk (34), alongside pharmacological interventions to promote erythropoiesis (e.g., iron, folic acid, and erythropoietin supplementation) (35, 36). Nevertheless, blood transfusion remains the primary therapeutic modality for anemia in critically ill patients.

There is significant heterogeneity in tissue susceptibility to ischemia across different organ systems (37). When anemia strikes suddenly, the body tries to adapt but often cannot. The heart and lungs struggle to deliver extra oxygen. The blood’s ability to release oxygen also falls short. This failure puts vital organs in danger. The heart and the brain specifically face a high risk of damage from a lack of oxygen. Three body systems work together to deliver oxygen. These are the heart, the lungs, and the red blood cells. Transfusions quickly raise the amount of hemoglobin in the blood. This helps the body move oxygen more effectively. As a result, this treatment helps stop organ failure and shock. It remains a key part of care to save lives (38, 39); patients with chronic conditions often develop enhanced tissue tolerance through metabolic adaptations (40, 41); in these scenarios, blood transfusion may carry risks of circulatory overload or adverse transfusion reactions rather than providing benefit (2). Conversely, in patients experiencing acute exacerbation of chronic diseases, clinicians must be vigilant regarding potentially exhausted compensatory reserves, which may render tissues significantly more sensitive to ischemia. Even a marginal decline in hemoglobin levels may precipitate acute myocardial ischemia or multi-organ dysfunction; therefore, clinical decision-making should be guided by tissue perfusion markers–such as lactate and central venous oxygen saturation (ScvO_2_)–rather than relying solely on a single hemoglobin threshold (38, 41–43).

### 4.1 Risks and benefits of transfusion

All medical interventions and drug treatments require the balance of risks versus benefits, including but not limited to blood transfusion. Theoretically, the administration of packed red blood cells aims to improve oxygen delivery to tissues and contribute to the maintenance of physiological homeostasis (18). Clinical studies have demonstrated that transfusion is associated with reduced mortality in specific subgroups of ICU patients, notably patients aged 66–80 years, those undergoing non-cardiac surgery patients, individuals with high SOFA or SAPS II scores, and those diagnosed with severe sepsis (44).

However, while allogeneic blood transfusion carries inherent risks, the safety landscape has evolved fundamentally over the past two decades. Driven by the universal implementation of nucleic acid amplification testing (NAT) and rigorous donor screening, the risk of transfusion-transmitted viral infections has been virtually eliminated in high-income nations. This advancement has significantly shifted the overall risk-benefit calculus of RBC transfusion toward a more favorable profile compared with previous eras (45–48). Consequently, with infectious risks minimized, the primary focus of clinical vigilance in the ICU has transitioned toward noninfectious complications, including allergic reactions, acute hemolytic reactions, and transfusion-associated circulatory overload (TACO). Furthermore, it has been linked to an increased incidence of stroke, prolonged hospital stay, and higher rates of sepsis among critically ill patients. Additional serious complications may include transfusion-related acute lung injury, onset or exacerbation of acute respiratory distress syndrome (ARDS), delayed extubation, and an increased likelihood of reintubation (49).

In the ICU, the identification of transfusion-associated complications is paramount yet fraught with diagnostic challenges. Given that critically ill patients frequently present with underlying cardiopulmonary dysfunction, their clinical manifestations are often indistinguishable from the signs of adverse transfusion events (50). Epidemiological data indicate that the incidence of adverse transfusion reactions is significantly elevated in ICU patients compared to those in general ward (50). Notably, transfusion-associated circulatory overload (TACO) and transfusion-related acute lung injury (TRALI) warrant particular clinical vigilance due to their high prevalence and severe clinical sequelae.

1. Transfusion-associated circulatory overload (TACO)

Transfusion-associated circulatory overload represents the most prevalent serious adverse transfusion reaction encountered in clinical practice. While the incidence in the general adult inpatient population is approximately 1%, the pooled incidence among adult ICU patients reaches as high as 5.5% (51). Although definitions vary slightly across the literature, according to National Healthcare Safety Network (NHSN) criteria, a diagnosis of TACO requires the new onset or acute exacerbation of at least three of the following indicators within 6 h post-transfusion: respiratory distress, elevated brain natriuretic peptide (BNP or NT-pro-BNP) levels, increased central venous pressure (CVP), evidence of left heart failure, positive fluid balance, or pulmonary edema (52).

2. Transfusion-related acute lung injury (TRALI)

Transfusion-related acute lung injury has historically been considered the leading cause of transfusion-related morbidity and mortality. A retrospective study by Gajic et al. confirmed that blood transfusion serves as an independent risk factor for acute lung injury (ALI) in mechanically ventilated patients (53). Clinical manifestations of TRALI typically include dyspnea, tachypnea, and hypoxemia, occasionally accompanied by rigors, tachycardia, fever or hypothermia, and hemodynamic instability (manifesting as hypotension or hypertension). Chest radiography typically reveals bilateral interstitial infiltrates; however, this finding is non-specific and frequently challenging to differentiate from pulmonary edema secondary to volume overload. The diagnosis of TRALI is primarily predicated on clinical presentation, radiographic features, and a temporal correlation with transfusion (typically occurring within 6 h, with delayed onset up to 72 h occasionally reported). Differential diagnosis must rigorously exclude other acute transfusion reactions with mimicking clinical features, such as TACO, septic transfusion reactions, and allergic reactions (52).

In conclusion, given the substantial evidence indicating that transfusion may precipitate serious adverse outcomes, there is an urgent need to shift away from empirical transfusion practices that lack a solid evidence-based foundation.

Investigators have recently developed and validated a robust risk prediction score for RBC transfusion, utilizing data from 42,435 patients undergoing cardiopulmonary bypass cardiac surgery. This risk stratification tool enables preoperative categorization of patients into low, medium, or high-risk groups for RBC transfusion (54). Such an approach allows for the quantitative assessment of transfusion-associated risks, thereby advancing clinical practice beyond qualitative enumeration and the mere listing of risk factors.

3. Transfusion-transmitted infections (TTIs)

Transfusion-transmitted infections (TTIs) are defined by the transmission of pathogens from donor to recipient through blood components, resulting in subsequent infection or clinical disease. Although the risk of viral transmission in high-income nations is now negligible–attributed to the shortened diagnostic “window period”–with estimated residual risks for HIV, HBV, and HCV of less than one per million units, global disparities in blood safety remain a critical concern (45–48). In resource-limited settings, the prevalence of TTIs is significantly higher, driven by variable access to advanced screening technologies and the endemicity of specific pathogens. A salient example is malaria (*Plasmodium* species), which continues to pose a substantial challenge to blood safety in endemic regions (55). Furthermore, the blood supply remains vulnerable to emerging and re-emerging pathogens, such as Oropouche virus and Zika virus (56). Consequently, despite the modernization of blood banking, clinicians must maintain vigilance regarding regional infectious epidemiology and the potential transmission of novel pathogens.

### 4.2 Current transfusion strategies

Blood transfusion is a vital intervention in intensive care, necessitating a comprehensive evaluation of risks and benefits to guide clinical decision-making. In May 2010, the World Health Organization advocated for the implement Patient Blood Management (PBM) protocols for surgical patients starting from the preoperative phase. Amidst the ongoing global advancement of PBM initiatives (57), modern transfusion medicine has evolved toward evidence-based individualized treatment approaches. PBM is founded upon three core pillars, which are (1) the optimization of the red blood cell capacity, (2) the minimization of the blood loss and (3) elevation of physiological tolerance to anemia (58–61).

Patient Blood Management programs have witnessed widespread adoption across healthcare institutions. Current clinical practice primarily employs two strategic paradigms: the liberal strategy, which aims to maintains hemoglobin levels at 9–10 g/dL, and the restrictive strategy, which targets a lower threshold of 7–9 g/dL. However, it is imperative to recognize that these strategies represent experimental constructs rather than the exclusive modalities of clinical care. The specific definitions and thresholds characterizing restrictive and liberal arms exhibit significant heterogeneity across trials, complicating the formulation of uniform recommendations. Furthermore, recent evidence, including studies from Korea and global surveys, indicates a divergence between these fixed trial protocols and real world practice. Rigid adherence to such specific transfusion strategies is not prevalent in routine clinical care across North America, Europe, and Asia. Consequently, transfusion decisions in the ICU should not rely solely on static thresholds but must be grounded in continuous clinical evaluation and dynamic adjustment (3, 62, 63). For postoperative ICU patients, anemia correction to ensure adequate tissue oxygenation must be balanced with rational blood resource utilization and ethical principles avoiding over-transfusion (64). Consequently, the determination of transfusion thresholds should be grounded in continuous clinical evaluation and dynamic adjustment, integrated within the comprehensive framework of institutional blood management programs.

**FIGURE 2:**
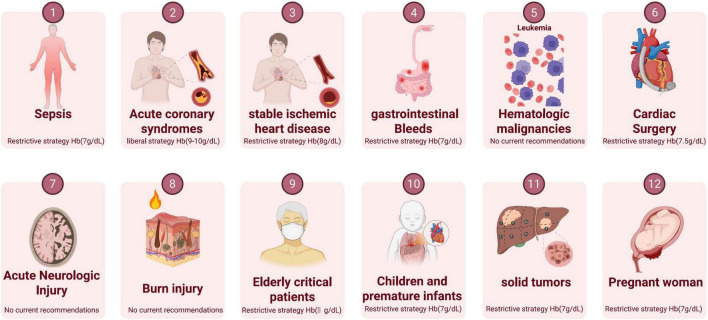
Summary of red blood cell transfusion thresholds in common ICU conditions.

The transfusion of packed RBCs represents a pivotal therapeutic intervention in the ICU, necessitating a tailored approach for specific patient populations (Figure 2). The paradigm shift toward a restrictive transfusion strategy was primarily driven by two seminal, albeit controversial, randomized controlled trials: the Transfusion Requirements in Critical Care (TRICC) trial (38) and the Functional Outcomes in Cardiovascular Patients Undergoing Surgical Hip Fracture Repair (FOCUS) trial (65). While the TRICC trial initially indicated the non-inferiority of a restrictive strategy in the general ICU population, it was the FOCUS trial–enrolling over 2,000 patients with cardiovascular risk factors–that fundamentally influenced global clinical practice and hospital policies. Notably, data from the FOCUS trial served as the cornerstone for the influential 2023 AABB International Guidelines, which strongly advocate for restrictive thresholds. These findings precipitated the gradual adoption of restrictive transfusion protocols over the subsequent decade, as demonstrated by randomized controlled trials performed in various clinical environments (Table 2). However, these landmark studies remain subject to significant debate. Critics argue that their study designs, which compared two fixed hemoglobin triggers without a “usual care” control arm, may have obscured safety signals and failed to reflect the complexity of real-world titrated care (66–70).

**TABLE 2:** Key studies of restrictive versus liberal transfusion strategies by patient population.

References	Study design	Patient	Restrictive strategy (Hb or HCT)	Liberal strategy (Hb or HCT)	Primary outcome and results
**General ICU**					
Hébert et al. (38) Carson et al. (65) Jiménez et al. (71)	Multicenter RCT	ICU patients *n* = 838	7 g/dL	10 g/dL	No difference in 30-days all-cause mortality (18.7% restrictive vs. 23.3% liberal; *p* = 0.11).
	Multicenter RCT	High-risk patients after hip surgery *n* = 2016	8 g/dL	10 g/dL	No significant difference was observed in the composite outcome of death or inability to walk independently at 60-days follow-up (OR 1.01; 95% CI 0.84–1.22), nor in in-hospital mortality, acute coronary syndrome, or other functional recovery measures between restrictive and liberal strategies.
	Meta-analysis	RCTs among adult ICU (13 studies)	7 g/dL	9–10 g/dL	No significant difference was observed in 30-days mortality (OR 1.02; 95% CI 0.83–1.25; I^2^ = 49%), nor in 90-days or 180-days mortality, hospital/ICU length of stay, dialysis requirement, or ARDS incidence between restrictive and liberal strategies.
**Sepsis**					
Holst et al. (4) Bergamin et al. (74)	Multicenter RCT	Septic shock *n* = 1005	7 g/dL	9 g/dL	No difference in 90-days mortality (43.0% vs. 45.0%; *p* = 0.44), incidence of ischemic events, severe adverse reactions, or proportion of patients requiring life support.
	Single-center RCT	Cancer patients with septic shock *n* = 300	7 g/dL	9 g/dL	No significant difference was observed in 28-days mortality (45% vs. 56%; HR 0.74, 95% CI 0.53–1.04; *p* = 0.08) or hospital/ICU length of stay between groups, despite more RBC units transfused in the liberal group (1 [0–3] vs. 0 [0–2] units; *p* < 0.001). However, significantly lower mortality was observed at 90 days with liberal strategy (59% vs. 70%; HR 0.72, 95% CI 0.53–0.97; *p* = 0.03)
**ACS**					
Cooper et al. (75) Ducrocq et al. (76) Carson et al. (78) Simon et al. (79)	Multicenter RCT	Acute MI *n* = 45	24%	30%	2.7% higher mean daily hematocrit in the liberal vs. restrictive group (*p* < 0.001) and a greater mean number of RBC units transfused in the liberal group (2.5 vs. 1.6; *p* = 0.07).
	Multicenter RCT	Acute MI *n* = 668	8 g/dL	10 g/dL	A 3.0% reduction in major adverse cardiovascular events [MACE; comprising all-cause death, stroke, recurrent myocardial infarction (MI), or ischemic revascularization] risk was observed in the restrictive versus liberal group at 30 days (95% CI −8.4% to 2.4%), with a relative risk of 0.79 (one-sided 97.5% CI 0.00–1.19) for the primary outcome, meeting the pre-specified non-inferiority criteria.
	Multicenter RCT	Acute MI *n* = 3504	7 or 8 g/dL	10 g/dL	A higher incidence of myocardial infarction (MI) or all-cause mortality was observed in the restrictive group compared with the liberal group within 30 days (16.9% vs. 14.5%; hazard ratio [HR] = 1.15, 95% confidence interval [CI]: 0.99–1.34; *p* = 0.07), however, secondary analyses indicated a reduced risk of cardiac death with the liberal strategy, suggesting a probability of clinical benefit.
	Multicenter RCT	Acute MI *n* = 3504	7 or 8 g/dL	10 g/dL	No difference in all-cause mortality at 6-months follow-up. 52% higher risk of cardiac death in the restrictive vs. liberal group (9.0% vs. 6.1%; *p* < 0.001).
**CVD**					
Docherty et al. (81) Ali et al. (82) Kougias et al. (83)	Meta-analysis	CVD (11 Multicenter RCT)	7–9.7 g/dL	9–11.3 g/dL	1.15 pooled hazard ratio for transfusion threshold and 30-days mortality (95% confidence interval [CI]: 0.88–1.50, *p* = 0.50), with very low heterogeneity (I^2^ = 14%).
	Single-center RCT	Non-cardiac surgery *n* = 92	8 g/dL	10.4 g/dL	Higher postoperative hemoglobin levels in the liberal vs. restrictive group (*p* < 0.05). Higher incidence of all-cause mortality, myocardial infarction (MI), or unplanned coronary revascularization in the liberal vs. restrictive group (16% vs. 8%).
	Multicenter RCT	High cardiac risk *n* = 1428	7 g/dL	10 g/dL	No significant difference in primary composite outcome (9.1% Liberal vs. 10.1% Restrictive; RR 0.90). However, restrictive strategy significantly increased non-MI cardiac complications (9.9% vs. 5.9%), mainly heart failure.
**Severe acute gastrointestinal bleeds**					
Odutayo et al. (88)	Meta-analysis	Acute upper gastrointestinal bleeding (5 RCT)	7–8 g/dL 21%	9–10 g/dL 28%	Fewer red blood cell transfusion units in the restrictive vs. liberal group (mean difference = −1.73, *p* < 0.0001). There was a lower risk of all-cause mortality (*p* = 0.03) and rebleeding (*p* = 0.004) in the restrictive group. No statistically significant differences in risk of ischemic events or among subgroups (cirrhosis/non-variceal bleeding) between the two groups.
**Malignant hematologic**					
Radford et al. (90)	Meta-analysis	Malignant hematologic disorder or hemopoietic stem cell transplant (7 studies)	7–9 g/dL	8–12 g/dL	Very low-quality evidence: no significant difference in mortality within 31–100 days between the two groups. Very low-quality evidence suggests that a restrictive strategy has little impact on quality of life within 3 months. Low-quality evidence: no difference in incidence of bleeding, infection, or readmission rates between the two groups. Moderate-quality evidence: no statistically significant difference in length of hospital stay between the two groups.
**Cardiac surgery**					
Murphy et al. (96) Mazer et al. (97) Garg et al. (98)	Multicenter RCT	Cardiac surgery *n* = 2007	7.5 g/dL	9 g/dL	Slightly higher incidence of severe infection or ischemic events within 3 months in the restrictive vs. liberal group (35.1% vs. 33.0%; *p* = 0.30). The significantly higher mortality rate in the restrictive vs. liberal group (4.2% vs. 2.6%, *p* = 0.045).
	Multicenter RCT	Cardiac surgery *n* = 5243	7.5 g/dL	8.5–9.5 g/dL	Lower incidence of all-cause mortality, myocardial infarction (MI), stroke, or new-onset renal failure in restrictive vs. liberal group [11.4% vs. 12.5%; absolute risk difference (ARD) = −1.11%, 95% confidence interval (CI) −2.93 to 0.72; odds ratio (OR) = 0.90, 95% CI 0.76–1.07, *p* < 0.001, meeting non-inferiority criteria]. Restrictive strategy is non-inferior to liberal strategy in high-risk patients undergoing cardiac surgery.
	Multicenter RCT	Cardiac surgery *n* = 4531	7.5 g/dL	8.5–9.5 g/dL	No difference in incidence of AKI between the two groups (27.7% vs. 27.9%). No difference among patients with concurrent chronic kidney disease (CKD) (33.6% vs. 32.5%).
**Head injury**					
McIntyre et al. (102) Turgeon et al. (103) Taccone et al. (105)	Multicenter RCT	Moderate to severe closed head injury *n* = 67	7 g/dL	9 g/dL	No difference in 30-days mortality between groups (17% restrictive vs. 13% liberal; *p* = 0.64).
	Multicenter RCT	Moderate or severe traumatic brain injury *n* = 200	7 g/dL	10 g/dL	No difference in Glasgow Outcome Score 6 months post-injury (42.5% restrictive vs. 33.0% liberal; *p* = 0.28). Lower thromboembolism incidence in restrictive (8.1%) vs. liberal group (21.8%; *p* = 0.009).
	Multicenter RCT	Acute brain injury *n* = 820	7 g/dL	9 g/dL	A significant difference in the incidence of neurological adverse outcomes during 180-days follow-up (72.6% restrictive vs. 62.6% liberal; *P* = 0.002).
**Burn injury**					
Palmieri et al. (106)	Multicenter RCT	Burn patients (≥20% total body surface area) *n* = 345	7 g/dL	10 g/dL	No difference in incidence of bloodstream infection between groups (24% restrictive vs. 24% liberal; *p* = 0.904). Lower red blood cell transfusion volume in the restrictive vs. liberal group (8 U vs. 16 U; *p* < 0.0001).
**Older critically ill patients**					
Walsh et al. (108)	Multicenter RCT	Mechanically ventilated patients *n* = 100	7 g/dL	9 g/dL	No difference in organ dysfunction, duration of ventilation, infection, or incidence of cardiovascular complications between the two groups; higher trend of 180-days mortality in liberal vs. restrictive group (55% vs. 37%; *p* = 0.073).
**Children**					
Lacroix et al. (109) Cholette et al. (110)	Multicenter RCT	Critically ill *n* = 673	7 g/dL	9.5 g/dL	Reduced blood transfusion volume by 44% in the restrictive vs. liberal group (*p* < 0.001). Comparable incidence of multiple organ dysfunction syndrome (MODS) between restrictive and liberal groups (absolute risk reduction: 0.4%, 95% CI −4.6∼5.4); no significant difference in other outcomes.
	Single-center RCT	Single-ventricle physiology *n* = 60	9 g/dL	13 g/dL	Reduced frequency of blood transfusion, donor exposure, and potential risks in restrictive vs. liberal group in children.
**Other tumor without Malignant hematologic**					
de Almeida et al. (111) Bergamin et al. (74) Chapalain et al. (112)	Single-center RCT	Surgical oncology patients *n* = 198	7 g/dL	9 g/dL	Higher 28-days mortality and morbidity rates in the restrictive vs. liberal group (28.6% vs. 19.6%; *p* = 0.012).
	Single-center RCT	Adult cancer patients with septic shock *n* = 300	7 g/dL	9 g/dL	No significant difference in overall mortality between the restrictive and liberal groups (56% vs. 45%; HR = 0.74, 95% CI 0.53–1.04; *p* = 0.08). Lower 90-days mortality in liberal vs. restrictive group (59% vs. 70%; *p* = 0.03).
	Multicenter RCT	High-risk oncologic surgery *n* = 30	7.5 g/dL	9.5 g/dL	No significant impact on functional recovery in critically ill patients following major oncologic surgery between two transfusion strategies (liberal 26.7%: restrictive 20%; *p* = 1).

ACS, acute coronary syndrome; CVD, coronary vascular disease; MI, myocardial infarction; RCT, randomized controlled trial.

Recommendations from the 2018 Frankfurt Consensus Conference advocate that restrictive strategies should be applied primarily in hemodynamically stable patients (13). Multiple randomized controlled trials (RCTs) and meta-analyses have revealed no significant differences in short-term mortality or ICU length of stay between liberal and restrictive strategies (71). No statistically significant differences were observed regarding 90-days or 180-days mortality, duration of hospital or ICU stay, necessity for renal replacement therapy, or occurrence of ARDS. In 2025, American College of Chest Physicians Clinical Practice Guideline suggest that restrictive transfusion has been shown to reduce blood usage and transfusion-related complications without compromising safety in many critically ill populations, with the notable exception of those with acute coronary syndromes (10).

Drawing upon robust evidence from one of the largest international prospective cohorts to date, the study provides an extensive overview of clinical reasons and physiological triggers guiding transfusion decisions (3). These findings hold significant implications for the individualized assessment of hemodynamically stable critically ill patients, thereby influencing transfusion strategies for this population.

## 5 Updates of transfusion strategies for ICU disorders

Interpreting transfusion trial data requires tracing the historical origins of hemoglobin thresholds. For much of the 20th century, clinical practice adhered to the empirical “10/30 rule,” which mandated transfusion when hemoglobin fell below 10 g/dL or hematocrit below 30% (72). However, growing recognition of transfusion-related complications prompted a critical reassessment of this dogma. Driven by this paradigm shift, the landmark TRICC trial pragmatically designed two distinct intervention arms to achieve sufficient statistical separation: a liberal arm (10 g/dL) reflecting traditional practice, and a restrictive arm (7 g/dL) probing the limits of physiological tolerance (38, 73). Consequently, it must be acknowledged that these thresholds are pragmatic experimental constructs rather than precise physiological markers. While statistically robust, this dichotomous design may fail to capture the clinical complexity of “titrated care,” in which thresholds are dynamically adjusted based on age, cardiovascular reserve, and symptom burden (11). Until large-scale trials evaluate such individualized strategies against fixed protocols, the generalizability of a uniform restrictive approach remains a subject of debate.

### 5.1 Sepsis

Sepsis represents a life-threatening condition characterized by dysregulated host response to infection. Maintaining adequate organ perfusion is a cornerstone of clinical management.

The foundational guidance relies on robust evidence from major randomized trials. The earlier TRICC trial subgroup analysis (1999) suggested no mortality difference between restrictive and liberal strategies (38). Crucially, the large-scale randomized trial (2014) (*n* = 998) conducted in 2014 involving patients with septic shock demonstrated no significant differences in 90-days mortality, requirement for life-supporting therapies, or incidence of ischemic events between liberal (9 g/dL) and restrictive (7 g/dL) transfusion strategies (4).

While a single-center trial by Bergamin et al. observed a survival benefit with a liberal strategy in a specific cohort of septic oncology patients, this finding remains an outlier. It contradicts the larger body of evidence, and the results (liberal 9 g/dL vs. restrictive 7 g/dL) should be interpreted with caution due to the single-center design and specific high-risk population (74).

Consistent with the high-quality evidence from the TRISS trial, the 2021 Surviving Sepsis Campaign guidelines recommend a restrictive transfusion approach for adults with sepsis, maintaining hemoglobin between 7 and 9 g/dL, identifying this as a strong recommendation with moderate quality of evidence (14).

### 5.2 Acute coronary syndromes

Acute coronary syndrome (ACS) is triggered by an abrupt reduction in coronary blood flow, resulting in insufficient myocardial oxygen supply. High-risk patients frequently have a history of anticoagulant or antiplatelet use, which predisposes them to gastrointestinal hemorrhage and may subsequently lead to anemia. Furthermore, anemia exacerbate myocardial ischemia and heightens the susceptibility to infarction in patients with underlying coronary artery disease. In this context, blood transfusion serves as the primary intervention for rapid hemoglobin correction.

The CRIT trial (2011) reported that the incidence of a composite endpoint–comprising in-hospital mortality, recurrent infarction, or worsening heart failure–was significantly lower in the restrictive transfusion group (hematocrit < 24%) relative to the liberal transfusion group (hematocrit < 30%) (13% vs. 38%) (75). The 2016 REALITY trial initially corroborated these observations, demonstrating a marginally lower rate of 30-days MACE in the restrictive group (threshold 8 g/dL) compared to the liberal group (10 g/dL) (11% vs. 14%), alongside reduced all-cause mortality rates (5.6% vs. 7.7%, respectively) (76). However, extended monitoring revealed distinct safety signals. The 1-year follow-up study published in Circulation indicated that the restrictive strategy failed to achieve non-inferiority regarding the primary composite endpoint. Notably, a *post hoc* analysis identified a significant increase in MACE between day 30 and 1 year in the restrictive arm (hazard ratio, 1.44; 95% CI, 1.01–2.03). This late accrual of adverse events may reflect delayed harm attributable to persistent anemia, potentially predisposing patients to increased risks of sudden death or arrhythmias (77).

Subsequently, the larger-scale MINT trial (2023) enrolled three to four times as many participants as earlier studies (78). Its results indicated a 30-days rate of myocardial infarction or mortality of 16.9% in the restrictive group (7–8 g/dL), compared to 14.5% in the liberal group (10 g/dL). Although the estimated effect favored the liberal strategy, the primary composite endpoint did not reach statistical significance (*p* = 0.07). However, secondary analyses indicated a strong signal for benefit. Cardiac mortality was notably higher in the restrictive strategy group, which exhibited a 52% increased risk of cardiac death at 6 months. The 6-months hazard of cardiac death was 52% greater in the restrictive group compared with the liberal group [9.0% vs. 6.1%; *P* < 0.001, driven mostly by events within 30 days (79)]. Therefore, while definitive superiority regarding the primary outcome was not statistically established, the MINT trial results suggest that a liberal transfusion strategy may be preferable in this population to mitigate the risk of adverse cardiac events (78, 79). Subgroup analyses further suggested that under a liberal strategy, patients with type 1 myocardial infarction experienced a greater reduction in 30-days mortality or recurrent infarction compared to those with type 2 infarction.

In 2024, the American College of Chest Physicians Clinical Practice Guideline broadly supports a liberal transfusion strategy (12), but additional large-scale trials are necessary to substantiate these evidence-based recommendations.

### 5.3 Stable cardiovascular disease

Patients with chronic cardiovascular disease and anemia generally experience a worse prognosis, although the precise underlying mechanisms remain incompletely elucidated. The prevailing consensus posits that anemia may exacerbate cardiovascular risk by reducing myocardial oxygen supply while concurrently augmenting cardiac oxygen demand (80). Therefore, establishing a safe and precise hemoglobin threshold for transfusion is critical to minimizing procedure-related risks.

A meta-analysis (2016) involving patients with cardiovascular disease found no significant difference in 30-days mortality between restrictive (7–9.7 g/dL) and liberal (9–11.3 g/dL) transfusion strategies (81). However, the body of evidence indicating that restrictive transfusion may increase the risk of acute coronary syndromes remains limited. Notably, a single RCT (2024) involving high-risk cardiovascular patients undergoing non-cardiac surgery reported a significantly higher incidence of adverse cardiac events in the restrictive transfusion group (8 g/dL) relative to the liberal group (10.4 g/dL) (82).

Adding weight to these concerns, the recently published TOP trial (2025) involving 1,428 high-cardiac-risk veterans found that while the primary composite outcome was similar between strategies, the restrictive group (<7 g/dL) exhibited a significantly higher rate of cardiac complications, specifically heart failure and arrhythmias, compared to the liberal group (<10 g/dL) (83). These emerging data suggest that patients with underlying cardiac pathology may tolerate anemia poorly. In contrast, the observational InPUT study (*n* = 3,643) demonstrated that each additional RBC unit increased the odds of mortality, AKI, or ventilatory weaning failure, regardless of cardiac history. These findings suggest that the presence of cardiovascular disease does not mitigate inherent transfusion risks, such as volume overload and organ dysfunction (39).

Nevertheless, the updated 2023 AABB International Guidelines maintain the recommendation for a transfusion threshold of 8 g/dL for this population, consistent with prior versions (84). A restrictive strategy remains a viable considered in scenarios characterized by elevated troponin levels absent objective evidence of ongoing cardiac ischemia (12). However, this distinction between “stable” and “active” cardiovascular disease is increasingly scrutinized, as these conditions fundamentally represent a continuum of the same pathophysiology. Emerging evidence suggests that the unified risk of ischemia across this spectrum may be underestimated by current guidelines. While the original FOCUS trial (2011) is often cited to support the safety of restrictive thresholds, data subsequently clarified by the principal investigators in a 2018 meta-analysis (85) revealed a critical safety signal: MACE occurred nearly twice as frequently in the restrictive arm (32/631) as in the liberal arm (17/636; Risk Ratio 0.53, 95% CI 0.30–0.94). This significant increase corroborates the concern that restrictive thresholds may be unsafe for patients with underlying cardiovascular pathology. Furthermore, a recent comprehensive meta-analysis (86) demonstrated that restrictive strategies are associated with increased risks of MI and mortality, thereby challenging the rationale for applying a uniform 8 g/dL threshold to patients with significant cardiac pathology.

### 5.4 Severe acute gastrointestinal bleeds

In accordance with evolving guideline recommendations, our institution currently primarily admits patients with upper gastrointestinal bleeding (UGIB) who have an Glasgow-Blatchford Score (GBS) greater than 1 (87). Therapeutic interventions encompass gastrointestinal endoscopy, administration of proton pump inhibitors (PPIs), blood transfusion, and either surgery or transcatheter arterial embolization. However the diagnostic utility of hemoglobin levels in guiding transfusion decisions for patients with gastrointestinal hemorrhage remains limited, and optimal transfusion thresholds remain a subject of ongoing debate. Odutayo et al. (88) analyzed four published and one unpublished randomized trial (*n* = 1965), revealing that patients assigned to a restrictive transfusion strategy had lower rates of all-cause mortality and rebleeding compared with those assigned to a liberal strategy, along with reduced red blood cell utilization. Subgroup analyses showed no significant differences in outcomes among patients with ischemic heart disease, cirrhosis, or non-variceal bleeding, and there was no increased risk of ischemic events between the two strategies.

The 2021 American College of Gastroenterology (ACG) guidelines recommend a restrictive transfusion threshold of 7 g/dL (87). Concurrently, the European Society of Gastroenterology proposed an elevated threshold of 8 g/dL for patients with pre-existing cardiovascular disease (88). The 2023 ACG guidelines for lower gastrointestinal bleeding endorse a restrictive strategy (7 g/dL), extrapolating from evidence primarily derived from upper gastrointestinal bleeding studies (89).

### 5.5 Hematologic malignancies

The majority of heme malignancy care is outside of the ICU unless there is a severe complication, such as severe pneumonia, profound bone marrow suppression or hemophagocytic lymphohistiocytosis. Hemoglobin-supportive therapy represents a cornerstone in the clinical management of hematologic malignancies. A recent Cochrane systematic review (2024) evaluated the efficacy and safety of restrictive versus liberal transfusion strategies in patients undergoing intensive radiotherapy (90), incorporating seven small-scale studies (six randomized controlled trials, *n* = 560; one non-randomized trial, *n* = 84). The analysis revealed no significant differences between the restrictive (7–9 g/dL) and liberal (8–12 g/dL) transfusion strategies regarding all-cause mortality, quality of life, or infection rates within 31–100 days post-intervention. However, it is noteworthy that the overall certainty of evidence was rated as low.

Currently, two pivotal trials are underway: one targeting malignancy patients resuscitated from septic shock (91), and another focused on pediatric recipients of stem cell transplantation (92). A randomized trial conducted by DeZern et al. demonstrated that patients assigned to a restrictive strategy (7 g/dL) had a lower mean red blood cell utilization (by nearly four units) compared with those in the liberal strategy (8 g/dL), with no significant between-group differences in the rates of bleeding events or neutropenia (93). Given the current limitations in evidence, there remains a critical need for rigorously designed clinical trials to further elucidate the prognostic implications of restrictive transfusion strategies.

### 5.6 Cardiac surgery

Red blood cell transfusions are required in approximately half of all cardiac surgery patients, constituting this population as the largest consumer of blood products (94). However, intraoperative RBC transfusion largely mediated the effects of anemia on mortality (76%), intensive care unit stay (99%), and hospital stay (95). A study by Murphy et al. (96) reported no significant differences in the rates of severe infections or ischemic events within 3 months between restrictive (7.5 g/dL) and liberal (9 g/dL) transfusion strategies. Notably, a large-scale randomized controlled trial by Mazer et al. (97) demonstrated that a restrictive strategy (7.5 g/dL) was non-inferior to a liberal strategy (8.5–9.5 g/dL) regarding a composite endpoint of all-cause mortality, myocardial infarction, stroke, or new-onset renal failure. Similarly, Garg et al. (98) found that a restrictive threshold (7.5 g/dL) was associated with significantly lower red blood cell utilization compared with a liberal threshold, with no significant difference in the incidence of AKI between the two groups.

However, the application of a uniform transfusion trigger is increasingly challenged by evidence of physiological heterogeneity. In 2016, the AABB Clinical Practice Guidelines recommended a hemoglobin transfusion threshold of 8 g/dL for patients undergoing heart surgery, While earlier guidelines, such as the 2016 AABB (99) and 2018 Frankfurt Consensus (13), recommended fixed thresholds of 8 and 7.5 g/dL respectively, deeper analysis suggests that a “one-size-fits-all” approach may be inadequate. Notably, a significant interaction between age and transfusion strategy was observed in the TRICS III trial (*P* = 0.004). Older patients had lower rates of adverse events with a restrictive strategy, potentially due to susceptibility to transfusion-associated circulatory overload, whereas younger patients had better outcomes with a liberal strategy, likely reflecting higher metabolic oxygen requirements during recovery (97). This dichotomy argues against rigid adherence to a single hemoglobin number. Aligning with this nuance, the 2024 Guidelines on PBM for Adult Cardiac Surgery endorse a conservative baseline (considering transfusion at hemoglobin ≤ 7 g/dL during extracorporeal circulation and ≤8 g/dL postoperatively) but explicitly mandate further individualization based on dynamic oxygen supply indices (100).

### 5.7 Acute neurologic injury

Patients presenting with subarachnoid hemorrhage (SAH) and traumatic brain injury (TBI) frequently exhibit a poor prognosis, primarily attributed to the brain’s hypermetabolic state, which renders neural tissue highly susceptible to damage during hypoxia. Blood transfusion serves as a pivotal therapeutic intervention aimed at optimizing cerebral oxygen delivery and improving clinical outcomes in this context (101).

Early evidence from McIntyre et al. in patients with moderate-to-severe craniocerebral injuries reported no significant difference in 30-days mortality or ICU length of stay between restrictive (7 g/dL) and liberal (9 g/dL) strategies (102).

However, recent high-impact trials have provided conflicting but complementary insights. The large-scale RCT (2024) conducted by Turgeon et al. (*n* = 742), focusing specifically on traumatic brain injury (TBI), found no significant difference in long-term neurologic outcomes between liberal (10 g/dL) and restrictive (7 g/dL) strategies (103). Although the liberal group had a higher incidence of respiratory distress events.

In contrast, the TRAIN trial by Taccone et al. enrolled a broader population of acute brain injury (ABI), including SAH, and demonstrated that a liberal strategy (9 g/dL) was associated with a significantly reduced risk of unfavorable neurologic outcomes compared to a restrictive approach (62.6% vs. 72.6%) (104). This divergence suggests that study population heterogeneity plays a key role: while a restrictive strategy appears non-inferior in isolated TBI, a liberal strategy may offer neuroprotective benefits in a mixed ABI cohort (potentially driven by SAH benefits), warranting a nuanced approach based on specific pathology (103). Reflecting the complexity of these conditions, the 2019 European guideline on the management of major bleeding and coagulopathy following trauma recommended a restrictive transfusion approach for TBI based on preliminary evidence, while advising that decisions for SAH patients be individualized through comprehensive clinical assessment (105).

### 5.8 Burn injury

Aggressive fluid resuscitation, supplemented as necessitated by blood transfusion, serves as a pivotal component in the management of severe burn injuries. However, the distinctive pathophysiology characterizing burn injuries has contributed to a scarcity of high-quality clinical evidence guiding optimal transfusion practices in this population. A preliminary study (2017) by Palmieri et al. involving patients with moderately severe burns demonstrated that a restrictive transfusion strategy (threshold 7 g/dL) received significantly lower transfusion volumes compared with those in the liberal strategy group, with no significant between-group differences in the rates of organ dysfunction, mortality, or infection (106). Conversely, a retrospective analysis (2024) indicated that patients with severe burns and hemoglobin levels below 6.5 g/dL exhibited the highest mortality rate (107), a finding that has helped shape the design of subsequent randomized controlled trials (RCTs). Despite these contributions, a universally accepted transfusion threshold for burn patients remains undefined.

### 5.9 Elderly critically ill patients

Driven by global population aging, the ICU has increasingly become a primary setting for the management of geriatric critically ill patients. These individuals often present with anemia, dysregulated internal environment homeostasis, and diminished volume tolerance (84). Current WHO criteria define the elderly as individuals over 60 years of age. Hébert et al. demonstrated that the association between a restrictive strategy and reduced mortality was confined to patients under 55 years, whereas no such effect was observed in the geriatric population (38). However, this study notably lacked subgroup analyses specifically examining mechanically ventilated patients. Walsh et al. subsequently addressed this evidence gap by showing that a restrictive transfusion strategy (7 g/dL) was associated with significantly lower 180-days mortality compared to a liberal approach (9.5 g/dL) (37% vs. 55%), alongside more favorable overall prognostic outcomes (108). Although existing evidence generally favors restrictive transfusion practices, definitive threshold recommendations for this vulnerable population remain unclear.

### 5.10 Children and premature infants

Transfusion thresholds in critically ill pediatric patients have been relatively well-established. In a landmark study by Lacroix et al., a restrictive transfusion strategy (7 g/dL) was shown to significantly reduce red blood cell utilization without increasing the risk of multiple organ dysfunction syndrome (MODS) compared to a liberal strategy (9.5 g/dL) in children admitted to the ICU (109). These findings were corroborated by Cholette et al. in a study focusing on children with single-ventricle physiology (110). The 2023 AABB International Guidelines recommend the adoption of a restrictive transfusion strategy for critically ill children in general, while emphasizing that hemodynamically stable children with congenital heart disease necessitate individualized assessment (84).

### 5.11 Neoplasms

Solid malignancies are primarily characterized by local invasion, whereas hematological malignancies typically demonstrate systemic dissemination and disruption of hematopoiesis. These distinct mechanisms of anemia underlie the potential need for differing transfusion thresholds. According to de Almeida et al., patients assigned to a liberal transfusion strategy had significantly lower rates of 30-days mortality and postoperative complications (differences of 19.6% and 28.6%, respectively) compared with those assigned to a restrictive approach (111). Bergamin et al. (74) reported consistent findings, showing significantly higher 90-days mortality in the restrictive transfusion group (70% vs. 59%) when comparing thresholds of 7 g/dL versus 9 g/dL, though no differences were observed in ICU or hospital length of stay (75). This observed variance in outcomes may be attributable to the inclusion of critically ill oncology patients complicated by sepsis. In contrast, Chapalain et al. found that neither 30-days prognosis nor 90-days survival was significantly affected by transfusion strategy (7.5 vs. 9.5 g/dL) in high-risk oncology patients (112). Current evidence suggests that in patients with solid malignancies, a liberal transfusion threshold (9 g/dL) may be associated with more favorable survival outcomes, whereas hematologic malignancies show no significant prognostic differences between restrictive (7 g/dL) and liberal strategies, though the evidentiary basis remains limited.

Given the low quality of evidence and contradictory outcomes across these studies, the 2023 AABB International Guidelines maintain the recommendation for a restrictive transfusion strategy for critically ill oncology patients (85).

### 5.12 Pregnant women

The perioperative management of pregnant women presents unique challenges due to physiological adaptations, including hypervolemia-induced dilutional anemia and a blunted hemodynamic response (e.g., blood pressure, heart rate) to early blood loss (113).

Major Obstetric Hemorrhage (MOH) remains the leading cause of maternal mortality and morbidity worldwide (114). With approximately 500 million women of reproductive age affected by anemia globally, this condition not only compromises oxygen-carrying capacity and tolerance to hemorrhage but also correlates with an increased risk of postpartum hemorrhage (PPH) in proportion to anemia severity (115).

Given the extreme sensitivity of the placental unit to reductions in maternal oxygen supply, transfusion decision-making in obstetric patients requires a more proactive approach compared to that for general ICU populations.

Current guidelines vary in their emphasis on transfusion strategies: NATA (116) explicitly recommends a restrictive transfusion strategy with a hemoglobin threshold of <7 g/dL; while RCOG (117) and SOGC (118) endorse this threshold, they emphasize that decisions should not rely solely on hemoglobin values but must integrate hemodynamic status and clinical symptoms; conversely, ACOG (119), acknowledging that physiological compensation during pregnancy may mask signs of hemorrhage, advocates for early transfusion initiation rather than rigid adherence to specific thresholds. Notably, emerging evidence suggests that the transfusion of 1–2 units of red blood cells may be associated with a higher rate of severe maternal morbidity (120). In addition to packed red blood cells, tranexamic acid and fresh frozen plasma are recommended adjuncts for achieving hemostasis (116, 120, 121).

Although a restrictive transfusion threshold (Hb < 7 g/dL) has reached consensus, further randomized controlled trials are required to determine whether this threshold should be appropriately elevated in specific scenarios involving placental oxygen demands or impaired tissue perfusion secondary to preeclampsia.

## 6 Non-transfusion strategies and future directions

For critically ill patients who explicitly refuse allogeneic blood transfusion due to religious convictions or personal preference, clinical decision-making must transcend the traditional “hemoglobin threshold” paradigm and shift toward a sophisticated “Bloodless Medicine” strategy anchored in the three pillars of PBM (57–59):

1. Optimization of Endogenous Erythropoiesis

Pharmacological intervention serves as the cornerstone for maintaining hemoglobin homeostasis; clinicians should aggressively supplement critical hematopoietic substrates (e.g., intravenous iron, folic acid, and vitamin B12) (122, 123); although the use of Erythropoiesis-Stimulating Agents (ESAs) remains controversial in routine critical care, it represents a vital modality for stimulating bone marrow hematopoiesis in the specific context where transfusion is refused (124).

2. Minimization of Iatrogenic and Surgical Blood Loss

Given the substantial blood loss attributed to routine diagnostic phlebotomy in the ICU (median ∼40 mL/day), the implementation of blood conservation protocols–such as restricting sampling frequency and volume–can reduce iatrogenic blood loss by at least 25% (125, 126).

Furthermore, for surgical patients, intraoperative Cell Salvage and Acute Normovolemic Hemodilution (ANH) may serve as acceptable autologous blood conservation strategies for this specific population (127, 128).

3. Enhancement of Physiological Tolerance to Anemia

Predicated on the physiology of tissue oxygen delivery (DO2), when hemoglobin oxygen-carrying capacity is compromised, therapeutic emphasis must shift toward optimizing the remaining determinants of oxygen transport.

By augmenting cardiac output, maintaining arterial oxygen saturation, ensuring effective circulating volume, and improving microcirculatory perfusion, the risk of tissue hypoxia can be effectively mitigated (129).

## 7 Future directions

Future advancements in transfusion strategies are poised to evolve along three major trends: first, a transition from reliance on a solitary hemoglobin threshold toward a multidimensional dynamic assessment system that incorporates metrics such as sublingual microcirculation monitoring (130) and tissue oxygen metabolism visualization (131); second, the application of AI to leverage large-scale datasets (132), facilitating the development of personalized transfusion threshold models and the reduction of blood product wastage; and third, the development of novel blood substitutes such as nanomaterial-based hemoglobin oxygen carriers (133) and *ex vivo* expansion techniques for hematopoietic stem cells (134) that may transform conventional transfusion paradigms.

## 8 Summary

Red blood cell transfusion is a pivotal therapeutic intervention in the management of anemia and hemorrhagic shock among critically ill patients. It functions primarily to restore hemoglobin levels, maintaining hemodynamic stability, and enhancing oxygen delivery and utilization. Current evidence-based guidelines provide a scientific framework for transfusion indications; however, their application requires dynamic adjustment based on individual patient characteristics.

Clinical decision-making must be rigorously anchored in established transfusion protocols while integrating a comprehensive evaluation of the patient’s underlying pathology, organ functional reserve, and dynamic physiological markers such as ScvO_2_ and tissue oxygen metabolism parameters through a multidisciplinary collaborative approach.

Future research endeavors should prioritize two pivotal directions: first, the development of stratified transfusion strategies, such as the West China Perioperative Transfusion Scoring System (WCPTS), to facilitate precision transfusion and optimize blood resource allocation; and second, the implementation of RCTs across various disease subgroups (e.g., acute coronary syndromes, traumatic brain injury) to more precisely delineate optimal transfusion thresholds. Many RCTs have been performed, unclear how many more would help without real world evidence or other input.

The integration of novel monitoring modalities including microcirculation assessment and near-infrared spectroscopy along with artificial intelligence based clinical decision support systems, will serve to bridge the gap between evidence-based guidelines with individualized patient care.
